# Emerging Natural-Product-Based Treatments for the Management of Osteoarthritis

**DOI:** 10.3390/antiox10020265

**Published:** 2021-02-09

**Authors:** Maria-Luisa Pérez-Lozano, Annabelle Cesaro, Marija Mazor, Eric Esteve, Sabine Berteina-Raboin, Thomas M. Best, Eric Lespessailles, Hechmi Toumi

**Affiliations:** 1 Laboratory I3MTO, EA 4708, Université d’Orléans, CEDEX 2, 45067 Orléans, France; marialuisa.perezlozano@univ-paris13.fr (M.-L.P.-L.); annabelle.cesaro@univ-orleans.fr (A.C.); eric.lespessailles@chr-orleans.fr (E.L.); 2 Plateforme Recherche Innovation Médicale Mutualisée d’Orléans, Centre Hospitalier Régional d’Orléans, 14 Avenue de l’Hôpital, 45100 Orléans, France; 3 Center for Proteomics, Department for Histology and Embryology, Faculty of Medicine, University of Rijeka, B. Branchetta 20, 51000 Rijeka, Croatia; marija.mazor@medri.uniri.hr; 4 Service de Dermatologie, Centre Hospitalier Régional d′Orléans, 14 Avenue de l’Hôpital, 45100 Orléans, France; eric.esteve@chr-orleans.fr; 5 Institut de Chimie Organique et Analytique ICOA, Université d’Orléans-Pôle de Chimie, UMR CNRS 7311, Rue de Chartres-BP 6759, CEDEX 2, 45067 Orléans, France; sabine.berteina-raboin@univ-orleans.fr; 6 Department of Orthopedics, Division of Sports Medicine, Health Sports Medicine Institute, University of Miami, Coral Gables, FL 33146, USA; txb440@med.miami.edu; 7 Centre Hospitalier Régional d’Orléans, Institut Département de Rhumatologie, 45067 Orléans, France

**Keywords:** osteoarthritis, natural products, cartilage, bone

## Abstract

Osteoarthritis (OA) is a complex degenerative disease in which joint homeostasis is disrupted, leading to synovial inflammation, cartilage degradation, subchondral bone remodeling, and resulting in pain and joint disability. Yet, the development of new treatment strategies to restore the equilibrium of the osteoarthritic joint remains a challenge. Numerous studies have revealed that dietary components and/or natural products have anti-inflammatory, antioxidant, anti-bone-resorption, and anabolic potential and have received much attention toward the development of new therapeutic strategies for OA treatment. In the present review, we provide an overview of current and emerging natural-product-based research treatments for OA management by drawing attention to experimental, pre-clinical, and clinical models. Herein, we review current and emerging natural-product-based research treatments for OA management.

## 1. Introduction

Osteoarthritis (OA) is the most common degenerative musculoskeletal disease and is a leading cause of disability in the adult population [1]. OA is a whole-joint disease that is characterized by irreversible cartilage degradation; disruption of the tidemark, accompanied by angiogenesis and cartilage calcification; subchondral bone remodeling; osteophyte formation; mild-to-moderate inflammation of the synovial lining [2,3,4]. The most common risk factors for OA include age, prior joint injury, obesity, muscle atrophy, metabolic disorders, and mechanical stress [5,6]. The disease evolution is typically slow and can take years to develop, with resultant joint pain and stiffness, mobility limitations, and compromised quality of life. Despite the tremendous personal and societal burden of OA, there are no curative treatments available and most conventional therapies (medications, physiotherapy, mechanical devices) provide relatively short-term, unsustained relief of the symptoms [7,8,9,10,11].

Promise exists for emerging disease-modifying drugs in the management of OA patients that regulate cartilage metabolism, subchondral bone remodeling, synovial inflammation, and angiogenesis. Recently, the use of plant-derived natural products has increased because of their therapeutic value in bone health, which is attributable to their chondroprotective and osteoprotective properties [12,13]. Many of these natural products have been reported to have anti-inflammatory and antioxidant properties, anti-catabolic effects on chondrocytes, and inhibitory effects on osteoclast differentiation [14,15,16]. Accordingly, this review of natural-derived compounds that have shown promise in the treatment of OA highlights our current thinking for this novel approach.

## 2. Natural-Compound-Based Treatments for OA Therapy

Conventional pharmaceutical agents (steroids or non-steroids anti-inflammatory (NSAIDs) drugs) have small-to-moderate effects in patients with OA [7,9,11,17,18,19]. Accordingly, there is an increasing interest in identifying novel approaches, including the use of natural bioactive components that could promote joint health, and mitigate and/or reverse OA [20].

### 2.1. Alkaloids

#### Berberine

Berberine is an alkaloid (benzylisoquinoline) that is found in medicinal plants of the genera *Berberis*, such as *Berberis vulgaris*, and is usually found in the roots, rhizomes, and stems (Table 1) [21]. It has been reported that berberine has anti-osteoarthritic effects [21]. In vivo studies in two different OA animal models (collagenase- and surgically induced OA) have demonstrated that berberine has chondroprotective effects, which ameliorates cartilage degradation while inducing chondrocyte proliferation [22,23]. It has been shown that berberine inhibits chondrocyte apoptosis and cartilage degradation via activating AMPK signaling and suppressing p38 MAPK activity [24,25]. Berberine also decreases inflammation and cartilage degradation by modulating the host immune response through the inhibition of TLR4/NF-κB signaling [26]. Moreover, berberine has been associated with bone formation by promoting osteogenic differentiation via activation of Runx-2 and p38 MAPK and reducing osteoclast differentiation [27,28].

### 2.2. Flavonoids

#### 2.2.1. Apigenin

Apigenin is a flavonoid (4′,5,7-trihydroxyflavone) that is found in herbs (chamomile, thyme), fruits (orange), vegetable oils (extra virgin olive oil), and in plant-based beverages (tea, beer, and wine) (Table 2) [29]. This bioactive agent has already been used as therapeutic therapy against diabetes, cancer, Alzheimer’s disease, and OA [30,31]. Apigenin has anti-inflammatory properties through inhibiting IL-1β/NF-κB and TGFβ/Smad2/3 pathways in chondrocytes [32]. Park et al. have demonstrated that apigenin blocks cartilage degradation in in vitro and in vivo OA mouse models through Hif-2α inhibition and the consequent downregulation of MMP-3, MMP-13, ADAMTS-5, and ADAMTS-4 in articular chondrocytes [33]. Furthermore, apigenin has shown bone protective effects via modulating the gene expression of TGF-β1 and its receptors, BMP-2, BMP-7, ALP, and collagen type I in MG63 osteoblasts [34]. Apigenin also promotes osteogenic differentiation of human mesenchymal stem cells through the JNK and p38 MAPK pathways [35].

#### 2.2.2. Astragalin

Astragalin is a natural flavonoid (kaempferol 3-glucoside) found in various traditional medicinal plants, such as *Cuscuta chinensis*. Its antioxidant and anti-inflammatory therapeutic properties have led some to consider its potential as a therapeutic agent for OA patients [36,37]. According to Ma et al. [38], astragalin inhibits the IL-1β-stimulated activation of NF-κB and MAPK in the chondrocytes of patients with OA while suppressing inflammation and bone destruction in a mouse model of OA [38,39].

#### 2.2.3. Baicalein

Baicalein is a flavonoid (5,6,7-trihydroxyflavone) that is isolated from the roots of *Scutellaria baicalensis* and *Scutellaria lateriflora* and has medicinal properties, including neuroprotective, anti-oxidant, anti-fibrosis, and anti-cancer properties [40,41]. Recently, it has been demonstrated that baicalein has anti-catabolic and anti-apoptotic effects through inhibiting IL-1β induction in chondrocytes [42,43]. Another study showed that the intra-articular injection of medium and high doses of baicalein alleviated OA progression in a rabbit OA model, diminishing cartilage degradation, and showing a lower Mankin score [44]. Similarly, positive results were obtained on bone through the induction of osteoblast differentiation and inhibiting osteoclast differentiation [45,46].

#### 2.2.4. Chrysin

Chrysin is a flavonoid (5,7-dihydroxyflavone) that is found in various medicinal plants, such as *Scutellaria baicalensis* and *Passiflora caerulea*, but also in honey and propolis [47]. In human osteoarthritic chondrocytes, chrysin showed a suppressive effect on the IL-1β-induced inflammatory response, including the expression of inducible nitrous oxide synthase (iNOS), COX-2, MMP-1, MMP-3, MMP-13, ADAMTS-4, and ADAMTS-5 via the inhibition of NF-κB signaling and decreases in the concentrations of nitrous oxide (NO) and PGE2. Chrysin also inhibits the degradation of aggrecan and collagen-II [48]. In addition, chrysin attenuates the apoptosis and inflammation of stimulated human OA chondrocytes via the suppression of high-mobility group box chromosomal protein (HMGB-1) [49]. An osteoprotective effect was also observed under chrysin treatment via ERK/MAPK activation and the upregulating of Runx-2 and Osx expression [50,51].

#### 2.2.5. Genistein

Genistein is a flavonoid (isoflavone) and a phytoestrogen that is extracted from *Genista tinctoria*. It has been reported to have promising benefits in the treatment of several pathologies [52,53,54]. The anti-osteoarthritic activity of genistein is suggested to be due to the relationship between OA and altered estrogen metabolism [55]. Phytoestrogens have some estrogen activity and ameliorate menopausal symptoms, bone loss, and symptoms of OA [56,57]. In vitro, genistein suppresses catabolic effects of IL-1β-induced in human OA chondrocytes by targeting the Nrf2/HO-1 pathway, decreasing the expression of MMPs, nitric oxide synthase 2 (NOS2), and COX-2 [58]. In vivo, genistein attenuated cartilage degradation in two different OA animal models [58,59]. Furthermore, a positive effect on bone was obtained through enhanced osteoblastic differentiation and maturation via the activation of ER (estrogen receptor), p38 MAPK–Runx2, and NO/cGMP pathways [60,61,62]. It also inhibited osteoclast formation and bone resorption by inducing the osteoclastogenic inhibitor osteoprotegerin (OPG) and by blocking NF-κB signaling [60,63].

#### 2.2.6. Icariin

Icariin is a flavonoid (flavonoid glycoside) obtained from the genus *Epimedium.* The therapeutic potential of this natural compound in cartilage regeneration has been shown in both in vitro and in vivo studies [64,65]. In vitro, Icariin increases the secretion of extracellular matrix proteins, such as collagen type II and the expression of SOX-9, while decreasing the expression of MMPs via the activation of HIF-1α. In vivo, icariin enhances articular cartilage repair in mouse osteochondral-defective models [65]. It has been reported that icariin protects chondrocytes from lipopolysaccharide (LPS)-, IL-1β-, or TNF-α-induced inflammation. Apoptosis and extracellular matrix degradation was also observed via diminishing the expression of MMP-1, 3, 9, 13, COX-2, and iNOS, suppressing NF-κB signaling and activating the Nrf2/ARE pathway [66,67,68]. Icariin also demonstrates protective effects in bone metabolism. This compound can induce osteoblast proliferation, differentiation, and mineralization through estrogen-receptor-mediated ERK and JNK signal activation in the MC3T3-E1 osteoblastic cell line, resulting in an increased expression of differentiation markers, alkaline phosphatase (ALP), and collagen type I [69]. It has been demonstrated that icariin induces the miR-153/Runx2 pathway, which is involved in osteoblast differentiation [70]. Icariin also attenuates hypoxia-induced oxidative stress and apoptosis in osteoblasts [71]. In an in vivo OA mouse model, it was shown that icariin enhanced bone remodeling with a positive effect on subchondral bone and hyaline cartilage [72].

#### 2.2.7. Kaempferol

Kaempferol is a flavonoid (3,4′,5,7-tetrahydroxyflavone) that is derived from the rhizome *Kaempferia galanga* L. and can also be found in numerous common vegetables and fruits, including beans, broccoli, cabbage, grapes, strawberries, tomatoes, citrus fruits, and apples [73]. Kaempferol alleviates IL-1β-stimulated inflammation in rat OA chondrocytes by decreasing the production of PGE2 and NO and downregulating the expression of MMPs, ADAMTS-5, iNOS, and COX-2. These effects were all mediated through the inhibition of the MAPK p38 and NF-κB pathways [74,75]. It has been shown that kaempferol increased the osteoblast differentiation and mineralization, and increasing the expression of BMP-2, Runx-2, Osx, and collagen type I by activating Wnt/β-catenin signaling [76,77]. Another study revealed that kaempferol stimulated bone formation in part via the mTOR signaling pathway [78]. Kaempferol prevents osteoclast formation through MAPKs, c-Fos, and NFATc1 [76,79]. In addition, in vivo studies have reported that kaempferol decreased bone loss in ovariectomized mice [80,81].

#### 2.2.8. Luteolin

Luteolin is a flavonoid (3′,4′,5,7-tetrahydroxyflavone) that is present in herb vegetables and fruits, including *Salvia tomentosa*, *Chrysanthemum indicum*, *Artemisia asiatica*, broccoli, carrots, peppers, cabbages, parsley, thyme, peppermint, basil, and celery [82]. Luteolin has shown anti-inflammatory and anti-catabolic effects in chondrocytes through the inhibition of NF-κB signaling [83]. It diminishes the IL-1β-induced production of NO, PGE2, TNF-α, MMP-2, MMP-3, MMP-8, and MMP-9; downregulates the expression of COX-2, iNOS, MMP-1, MMP-3, MM-13, ADAMTS-4, and ADAMTS-5; inhibits the degradation of collagen type II [83,84,85]. Luteolin also protects chondrocytes from apoptosis by increasing Foxo3a expression via regulating the IRE1α pathway and miR-29a/Wnt/β-catenin signaling [86,87]. Its administration can also attenuate cartilage degradation and increase collagen type II expression in OA rats in vivo [83]. In vitro studies have demonstrated that luteolin upregulates the expression of osteoblastic differentiation markers, including TGF-β1, BMP7, Runx-2, ALP, Osc, Osx, and collagen type I [34,88,89]. It also has anti-oxidative and anti-apoptotic effects on osteoblasts, in part via the regulation of the ERK/Lrp-5/GSK-3β signaling pathway [90,91,92]. Furthermore, luteolin diminishes osteoclastic differentiation and function in vitro and in vivo, increasing the bone mineral density and content of trabecular and cortical bones in ovariectomized rats [93,94].

#### 2.2.9. Naringin

Naringin is a flavonoid (flavanone-7-*O*-glycoside) that is formed from the flavanone naringenin and the disaccharide neohesperidose, and is found in citrus fruits, such as grapefruit. Naringenin inhibited TNFα-, LPS-, and IL-1β-induced catabolic effects, diminishing the expression of MMPs, ADAMTS-4, and ADAMTS-5 via the suppression of the NF-κB pathway and caveolin–p38 MAPK signaling [95,96,97,98]. In vivo, naringin attenuates cartilage destruction via the suppression of inflammatory cytokines. Naringin also promotes bone formation via increased osteoblast proliferation and differentiation [99,100,101]. Mechanistically, this occurs through the increased expression and secretion of bone-formation-related genes including Osc, Runx-2, Osx, OPN, BMP-2, and collagen type I [102]. Naringin also inhibits osteoclast differentiation and maturation, therefore preventing bone loss [103].

#### 2.2.10. Puerarin

Puerarin is a flavonoid (isoflavone) that is found in several plants and herbs, such as the root of *Pueraria lobate* [104]. It reduces OA progression by inhibiting the pro-catabolic responses in chondrocytes [105]. It also has a negative effect on monocyte recruitment [106] and promotes bone formation through the estrogen receptor, p38 MAPK, ERK1/2–Runx2, and Wnt/β–catenin pathways [107,108]. Oral administration of puerarin in ovariectomized rats protected against a reduction in bone mineral density and content while improving femur trabecular bone structure [108]. The effects of puerarin on osteoblastic proliferation and differentiation are mediated by the inhibition of TRPM3/miR-204 expression and the activation of Runx-2 [109,110,111]. In ovariectomized rats, puerarin was shown to inhibit osteoblastogenesis through the downregulation of TRAP and RANKL [112]. The inhibition of RANKL osteoclastogenesis is mediated by the downregulation of CREB/PGC1β/c-Fos/NFATc1 signaling [113]. Furthermore, another study showed that puerarin inhibits osteoclastogenesis by suppressing RANKL-dependent and -independent autophagic responses [114].

#### 2.2.11. Silibinin/Silymarin

Silibinin, also known as silybin, is the major active flavonoid constituent of silymarin, which is an extract of milk thistle seeds (*Silybum marianum*), comprising approximately 50–70% of the extract [115,116]. It is also a phytoestrogen. Other flavonolignans, such as silychristin, isosilychristin, silydianin, and silimonin, are also present in silymarin. The anti-inflammatory properties of silymarin for OA treatment have been demonstrated using several protocols [115,116,117,118]. A study employing MIA-induced OA rats showed that silymarin exerts anti-inflammatory and antioxidant effects by diminishing the NO and IL-1β content in synovial tissue and attenuating cartilage degradation [119]. Another study demonstrated that silibinin inhibits the IL-1β-induced production of NO, PGE2, TNF-α, and IL-6; downregulates the expression of COX-2, iNOS, MMP-1, MMP-3, MMP-13, ADAMTS-4, and ADAMTS-5; diminishes the degradation of aggrecan and collagen type II in human OA chondrocytes through the suppression of PI3K/Akt and NF-κB signaling pathways [120]. Furthermore, treatment with silibinin prevented cartilage degradation and synovitis in an in vivo mice OA model. Silibinin also has osteoprotective properties, promoting osteoblastogenesis and inhibiting osteoclastogenesis [121,122]. In vitro experiments have shown that silibinin and silymarin induce osteoblast differentiation in MC3T3-E1 osteoblasts by increasing the expression of ALP, collagen type I, Runx-2, and BMP-2 [122,123]. It also promotes the osteogenic differentiation of human bone marrow stem cells via BMP signaling [124]. In addition, silibinin has antioxidant and anti-apoptotic effects in osteoblasts [125]. It has been reported that silibinin and silymarin suppress osteoclastic differentiation in RAW 264.7 osteoclasts, decreasing TRAP and cathepsin K induction induced by RANKL via disturbing TRAF6-c-Src signaling pathways and inhibiting femoral bone loss in ovariectomized mice [121,126].

#### 2.2.12. Wogonin

Wogonin is a flavonoid (O-methylated flavone) that is found in *Scutellaria baicalensis* as baicalein [127,128]. It has been reported that wogonin has chondroprotective effects, inhibiting IL-1β-induced catabolic markers, such as IL-6, COX-2, iNOS, MMP-3, MMP-9, MMP-13, and ADAMTS-4, while increasing the anabolic markers aggrecan and collagen type II in chondrocytes and cartilage explants [129,130,131]. These wogonin effects are mediated through the suppression of c-Fos/AP-1 and JAK/STAT signaling pathways and the activation of ROS/ERK/Nrf2 signaling pathways [129,131,132]. A recent study has shown that the utilization of tetrahedral framework nucleic acid/wogonin complexes alleviated inflammation in in vitro and in vivo OA models, preventing cartilage destruction and increasing bone mineral density [133]. Wogonin has also been shown to attenuate intervertebral disc degeneration [134].

### 2.3. Phenols

#### 2.3.1. Curcumin

Curcumin is a natural phenol (diferuloylmethane) that is responsible for turmeric’s yellow color and comes from the *Curcuma longa* root (Table 3). Anti-inflammatory, anti-oxidant, anti-apoptotic, and anti-catabolic effects were observed on chondrocytes under curcumin treatment. It inhibited the expression of the inflammation mediators IL-6, iNOS, and COX-2. It also blocked the expression of proteinases MMP-1, MMP-3, MMP-9, MMP-13, ADAMTS-4, and ADAMTS-5, and increased the expression of SOX-9 and production of collagen II, attenuating cartilage degradation [12,135,136,137,138]. These effects occur through the direct inhibition of 5-LOX and NF-κB, indirect inhibition of phospholipase A2 and COX-2, and activation of the Nrf2/ARE signaling pathway [135,136,137,138]. Chen et al. showed that curcumin also inhibited osteoblast apoptosis and promoted osteoblast differentiation, both in vitro and in vivo [139]. It increased the gene expression of Runx2, Osx, Osc, and collagen type I via the regulation of Wnt signaling [140,141]. The bioavailability of curcumin is a major challenge because it is inherently low in humans, but new formulations have enhanced the therapeutic efficacy of curcumin [142,143]. Furthermore, the use of curcumin in combination with other natural products, such as *Boswellia serrate*, gingerly, and pipeline, are being studied in several clinical trials to investigate whether their therapeutically synergy enhances their performance in OA treatment but the results showed no significant difference between each component separately or in combination [144,145,146].

#### 2.3.2. Gingerly/Ginger

Ginger is the rhizome of the *Zingier officinal* plant and has been commonly consumed as a spice and herbal medicine due to its anti-inflammatory properties. The major active component is the phenolic gingerly (6-gingerol) [147]. The efficacy and safety of ginger were evaluated in various studies [148]. Ginger extract has shown anti-inflammatory, antioxidant, and anti-apoptotic effects in IL-β-treated human chondrocytes via the activation of Nrf2 [149,150]. It also stimulated osteoblasts differentiation and inhibited IL-1β-induced osteoclasts differentiation in in vitro studies [151,152]. Randomized clinical trials have demonstrated that ginger extracts improved pain and mobility and reduced osteoarthritis inflammation in OA individuals [153,154]. The local application of ginger was also found to be effective at reducing symptoms of knee OA [155]. In addition, the synergistic effects of ginger with other natural products were also studied in patients with chronic OA, but the results did not show any significant enhanced effects [145,156,157].

#### 2.3.3. Oleuropein

Oleuropein is a phenolic compound (secoiridoid glycoside) that is present in green olive (*Olea europea*) and argan oil [158]. It has been reported that olive oil extract has beneficial effects in OA treatment [159]. An in vivo study demonstrated that oleuropein decreases the spontaneous development of OA in guinea pigs, reducing cartilage, osteophytes, and synovial OA scores [160]. It also inhibited IL-1β-induced inflammatory response in human OA chondrocytes in vitro by suppressing NF-κB and MAPK signaling pathways [161]. It suppresses the production of NO and PGE2 and decreased the expression of COX-2, iNOS, MMP-1, MMP-13, and ADAMTS-5. Furthermore, it has been shown that oleuropein does not stimulate osteoblast proliferation but increases the deposition of calcium and suppresses osteoclast formation and differentiation [162,163,164]. It also protected against bone loss in ovariectomized rats [165]. Yet, there is no clinical trial with this natural compound for OA; however, a randomized clinical trial with postmenopausal women showed that the consumption of a polyphenol extract from olive increases serum osteocalcin levels and improves serum lipid profiles [166].

#### 2.3.4. Resveratrol

Resveratrol is a stilbenoid (3,5,4′-trihydroxy-*trans*-stilbene), which is a type of natural phenol that is produced by several plants in response to injury and in fruits, such as red grapes, blueberries, raspberries, and mulberries [167,168]. Since it prevents degeneration and apoptosis, resveratrol has been strongly suggested to be a potential therapeutic agent for OA [169,170]. Resveratrol was demonstrated to inhibit IL-1β-induced catabolic effects in chondrocytes. It suppressed the expression of iNOS, MMP-3, MMP-1, MMP-13, ADAMTS-4, ADAMTS-5, and NO production by inducing SIRT-1 expression and inhibiting NF-κB signaling [171,172,173]. It also prevented IL-1β-mediated inflammation via TLR4 inhibition [174,175]. In vitro studies have demonstrated that these inhibitory effects of resveratrol are mediated via the activation of SIRT-1 by suppressing HIF-2 expression and inducing autophagy via the AMPK/mTOR pathway [171,176,177,178]. Preclinical models have shown that resveratrol treatment prevented OA progression, maintaining the structural homeostasis in cartilage and subchondral bone [173,178,179,180]. Resveratrol was demonstrated to exert bone protection through the suppression of osteoclast functions and the induction and differentiation of osteoblasts in both in vivo and in vitro studies. Resveratrol induced osteoblast differentiation by regulating autophagy and modulating the Sirt1/Runx-2/Fox-O1 and PI3K/AKT/mTOR signaling pathways, therefore, ameliorating bone loss in osteoporotic animal models [181,182,183,184]. It was also shown to induce osteoblastic MC3T3-E1 cells differentiation via the induction of the calcineurin/NFATc1 signaling pathway [185]. Resveratrol also inhibited RANKL-induced osteoclastogenesis via SIRT1 and FoxOs activation [186,187,188]. A clinical study on postmenopausal women showed that resveratrol supplementation reduces pain experience; thus, it was proposed as a potential treatment to reduce chronic pain in age-related osteoarthritic individuals [189]. Another pilot study demonstrated that the co-administration of resveratrol with meloxicam in patients with knee OA improves pain, functions, and associated symptoms compared with a placebo, yet it was superior in terms of safety and efficacy compared to meloxicam alone [190].

#### 2.3.5. Salvianolic Acid B

Salvianolic acid B (Sal B) is a major polyphenol constituent of the plant *Radix salvia miltiorrhiza*, which is commonly used in traditional Chinese medicine to cure pain [191]. It has been recently proposed as a potential therapeutic agent against OA that acts through the regulation of gene expression and the viability of chondrocytes [192]. It has been demonstrated that the pre-treatment of chondrocytes with Sal B followed by induction with IL-1β inhibited the overproduction NO and PGE2 and downregulated the expression of iNOS, COX-2, MMP-13, and ADAMTS-5 via the suppression of NF-κB [193]. This study also revealed that Sal B reduced cartilage degradation in an OA mouse model. Sal B was also found to stimulate osteoblastic differentiation in bone marrow stromal cells, upregulating the expression of Runx2, OPN, and Osx and stimulating mineralization through the activation of ERK signaling pathways [194]. In vivo, Sal B inhibited glucocorticoid-induced osteopenia. It enhanced bone thickness and bone mass by increasing the expression of BMPs, ALP activity, and collagen type I [194,195]. A pilot study in a rat tibia fracture model revealed that treatment with Sal B led to an enhancement in callus growth, histological scores, and post-fracture ALP activity, thus, accelerating early-stage fracture [196]. Furthermore, Sal B facilitates osteogenesis by targeting adipose tissue, reducing adipogenesis, and activating the MEK–ERK signaling pathway [197].

### 2.4. Polysaccharides

#### *Achyranthes bidentata* Extracts

*Achyranthes bidentata* is one of the most commonly used Chinese herbal medicines that is currently considered for the treatment of osteoarthritis (Table 4) [198]. This extract has shown chondroprotective effects in vitro, inducing chondrocyte proliferation via Wnt/β-catenin pathway activation and inhibiting apoptosis via the MAPK/Akt signaling axis [199,200]. Plant polysaccharides also have osteoprotective properties, suppressing osteoclastogenesis and bone resorption by inhibiting RANKL and promoting bone formation [201,202,203,204].

### 2.5. Terpenoids

#### 2.5.1. Andrographolide

This terpenoid (diterpenoid) is a natural component from *Andrographis paniculate*, a plant with medicinal properties, such as antioxidant, anti-inflammatory, and anti-arthritic properties (Table 5) [205,206,207,208]. A recent study showed the effectiveness and safety of andrographolide in reducing pain in individuals suffering from mild-to-moderate knee osteoarthritis [209]. It has been reported to inhibit the expression of MMPs and reduces oxidative stress injury in chondrocytes [210,211]. An in vivo mouse OA model study revealed that this compound alleviates cartilage damage via the miR-27-3p/MMP13 signaling axis [212]. It also exerts a pro-osteogenic effect via inducing bone formation by inhibiting NF-κB signaling, with this bioactive compound being a potential therapeutic target in OA [213,214].

#### 2.5.2. Astaxanthin

Astaxanthin is a carotenoid (tetraterpenoid) that is produced naturally in the microalgae *Haematococcus pluvialis* and can be found in animals who feed on the algae, such as salmon, red trout, and crustaceans [215]. It has therapeutic properties against rheumatoid arthritis and osteoarthritis [216,217,218,219,220]. In OA, astaxanthin has shown potent antioxidant and anti-inflammatory activities on cartilage due to the activation of Nrf2–ARE signaling in chondrocytes [221]. Astaxanthin also attenuated cartilage degradation in vitro and in vivo via blockade MAPK signaling [222]. Despite the fact that the effects of astaxanthin’s properties on OA bone remodeling have not yet been examined, it could be a good therapeutic target due to its effects on the suppression of bone loss in periodontitis and osteoporotic models [223,224].

#### 2.5.3. Aucubin

Aucubin is a terpenoid (iridoid glycoside) that is derived from diverse medicinal plants, including *Aucuba japonica* and *Eucommia ulmoides*. It has recently received increasing attention due to its pharmacological properties, including antioxidation, anti-inflammation, and osteoprotection [225]. In vitro studies showed that aucubin suppressed IL-1β-induced inflammation and matrix degradation and reduced oxidative stress by decreasing iNOS expression and the production of NO [226,227]. It has been reported that aucubin prevented OA progression in an in vivo mouse model and that the co-treatment with hyaluronic acid (HA) enhanced the anti-catabolic and anti-inflammatory effects of HA on OA chondrocytes [228,229].

#### 2.5.4. *Boswellia serrata*

*Boswellia serrata* is a plant that produces Indian frankincense and has two mains active terpenoid compounds, 11-keto-β-boswellic acid and acetyl-11-keto-β-boswellic acid [230]. The extracts of this plant have been clinically studied for osteoarthritis treatment, exerting anti-inflammatory activity and resulting in decreased pain and increased joint functionality [231]. *B. serrata* has been reported to have anti-inflammatory properties by inhibiting 5-LOX and TNF-α [232]. An in vitro model of cartilage degeneration showed that *B. serrata* diminished the catabolic effects mediated by IL-1α and oncostatin-M through inhibiting MMP-9 and MMP-13 transcription and reducing the levels of NO, PGE2, and COX-2 [233]. Its chondroprotective properties were confirmed in a mouse model of OA, showing antioxidative and anti-inflammatory effects [15,234]. Additionally, it has been reported that boswellic acids promoted osteoblast differentiation and suppressed osteoclastogenesis by inhibiting TNF-α and NF-κB signaling [235,236].

#### 2.5.5. Celastrol

Celastrol is a terpenoid (triterpenoid) that is isolated from the root extracts of *Tripterygium wilfordii* and *Celastrus regelii* [237]. Celastrol is an inhibitor of heat shock protein (HSP) 90β, which has chondroprotective effects. It has been reported that diminished IL-1β-induced catabolic effects in human osteoarthritic chondrocytes, such as the decrease expression of MMP1, MMP-3, MMP-13, iNOS, and COX-2 [238]. Using an in vivo OA rat model, it has been shown that celastrol suppresses apoptosis through the inhibition of the NF-κB signaling pathway and alleviates pain and cartilage damage via SDF-1/CXCR4 signaling [239,240]. Celastrol also has therapeutic effects on bone structure, where it prevented bone loss and bone microarchitecture degradation in a rat model of arthritis [241]. It has been shown that celastrol reduced the RANKL-induced expression of osteoclastic genes (TRAP, CTSK, CTR, and MMP-9) and transcriptional factors (c-Fos, c-Jun, and NFATc1), as well as the phosphorylation of NF-κB and MAPK in RAW 264.7 cells [242].

#### 2.5.6. Ginsenoside

Ginsenosides are a class of natural product triterpene saponins (terpenoid glycoside) that are found almost exclusively in the plant genus *Panax* (ginseng), which is used in traditional medicine [243]. Ginsenosides exhibit a large variety of subtypes with different chemical profiles and biological effects. It has been reported that ginsenosides Rg1, Rg3, Rg5, Rk1, Rf, Rd, Rc, and F4 have chondroprotective effects [244]. Ginsenoside Rb1 has antioxidative and anti-apoptotic effects in chondrocytes in vitro, stabilizing mitochondria and inhibiting caspase-3 through PI3K/Akt signaling [245,246,247]. It also suppresses IL-1β-induced effects on chondrocytes, decreasing MMP-1, MMP-13, iNOS, and COX-2 expressions and the concentration of PGE2, and promoting the expression of ACAN and collagen type II [248,249]. Ginsenosides, such as Rb1, Rg1, and Rg5 have alleviated inflammation and cartilage degradation in in vivo OA rat models [250,251,252]. Recent studies have reported the chondroprotective effect of different Panax plant extracts in vivo OA rat models, protecting chondrocytes from inflammation, senescence, and apoptosis, thus, attenuating OA progression [253,254]. Ginsenosides also have osteoprotective properties. Several studies have demonstrated that Rb1, Rh1, Rg3, and Rg5 stimulated osteoblast differentiation in vitro [255,256,257,258]. Furthermore, Rb1 and Rg3 inhibited osteoclastogenesis by suppressing RANKL-induced activation via modulating MAPKs and NF-κB pathways in vitro, but only Rg3 was able to alleviate bone mineral density loss in vivo [259,260].

#### 2.5.7. *Harpagophytum procumbens*

*Harpagophytum procumbens*, also known as devil’s claw, is a medicinal plant native to Africa that has been used as an analgesic for the treatment of degenerative diseases of the musculoskeletal system [261]. The bioactive components responsible for the anti-osteoarthritic effect are the iridoid glycosides (harpagoside, harpagide, and procumbide), which are found in a higher amount in the tubers and root [262]. An in vitro study showed that the pre-treatment of IL-1β-induced OA chondrocytes with harpagoside exerted some anti-inflammatory effects, inhibiting IL-6 and MMP-13 expression via the suppressing c-Fos/AP-1 activity [263]. Another study showed that harpagide improved bone properties, stimulating the differentiation of osteoblasts and suppressing the RANKL-induced differentiation of osteoclasts in an ovariectomized mouse model, thus, improving the recovery of bone mineral density and trabecular bone volume [264]. Furthermore, some human clinical studies showed that various *H. procumbens* tuber extracts improved clinical pain and movement limitation in individuals with knee and hip OA [265]. However, more studies are required to elucidate the therapeutic properties of *H. procumbens* in OA.

## 3. Conclusions

Osteoarthritis is a disease that is becoming more prevalent with the increase in the aging population. There are few conventional therapies that are available for the systematic treatment of OA and no treatment to prevent it. Unfortunately, all these therapies have significant adverse effects and are not adequate for long-term OA management. Therefore, the protective effects shown by natural products could be a potential alternative to conventional therapy. This review shows that natural compound supplementation plays an important role in the prevention of osteoarthritis. Various natural products have shown similar mechanistic properties, such as anti-inflammatory and antioxidant effects, on chondrocytes, inhibiting the cytokine-induced expression and catabolic activity of MMPs by inhibiting the NF-κB signaling pathway. Some phytochemicals have been shown to protect against cartilage degradation in preclinical studies. Natural products have also shown osteoprotective effects, upregulating the expression of various factors, such as Runx2, OPN, and Osx, in addition to the upregulation of the MAPK pathway and OPG/RANKL ratio. These regulations decreased bone resorption and enhanced osteoblastic activity and downregulation of the osteoclastic activity. Furthermore, some phytochemicals showed synergistic effects when explored in combination with other natural products or standard therapies. Although there are several bibliographical studies that show that some natural compounds are of interest in terms of fighting against inflammation or oxidation processes, as far as we know, there is no natural product that can prevent osteoarthritis or reverse it. The studies of these natural products from human clinical trials are still too few to be able to confirm their therapeutic effect at present. Therefore, the optimization of the formulation of natural products, and/or the combination of them, to combat and prevent osteoarthritis is a challenge.

## Figures and Tables

**Table 1:** Natural-alkaloid-based pharmacology therapy for osteoarthritis (OA).

Compound (Source)	Category	Structure	Therapeutic Target	Treatment	Ref.
Berberine (*Berberis vulgaris*)	Benzyl isoquinolin alkaloid	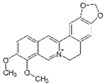	Activation of AMPK signaling and inhibition of p38 MAPK/NF-κB pathways in chondrocytes. Activation of p38 MAPK signaling in osteoblasts.	Anti-inflammatory, anti-apoptotic, and anti-degradation in cartilage; Induction of bone formation.	[22,23,24,25,26,27,28]

**Table 2:** Natural-flavonoid-based pharmacology therapy for OA.

Compound (Source)	Category	Structure	Therapeutic Target	Treatment	Ref.
Apigenin (chamomile, thyme, tea, extra virgin oil)	4′,5,7-Trihydroxyflavone	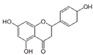	Inhibition of IL-1β-induced effects and NF-κB, Hif-2α, and TGFβ/Smad2/3 pathways in chondrocytes.	Anti-inflammatory effect, prevent cartilage degradation.	[32,33]
			Increases BMP-2, BMP-7, APL, and Col I in osteoblasts. Induces JNK and p38 MAPK pathways in osteoblasts.	Promotes osteoblastic differentiation.	[34,35]
Astragalin (*Cuscuta chinensis*)	Kaempferol 3-glucoside	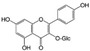	Inhibition of IL-1β/NF-κB and MAPK in chondrocytes.	Anti-inflammatory effect, suppresses bone destruction.	[38,39]
Baicalein (*Scutellaria baicalensis*)	5,6,7-Trihydroxyflavone	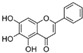	Inhibition of IL-1β-induced effects in chondrocytes. Increases secretion of GAG and Col II.	Anti-catabolic and anti-apoptotic effects.	[42,43,44]
			Increases osteoblast differentiation and inhibits osteoclast differentiation.	Attenuated OA in pre-clinical models. Inhibition of bone loss.	[45,46]
Chrysin (*Passiflora caerulea*, *Scutellaria baicalensis*)	5,7-Dihydroxyflavone	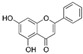	Inhibition of IL-1β/NF-κB induction. Dowregulates the expression of iNOS, COX-2, MMP-1, MMP-3, MMP-13, ADAMTS-4, ADAMTS-5, and HMGB-1 in chondrocytes. The level of NO, PGE2 decreases.	Anti-inflammatory and anti-apoptotic effects.	[48,49]
			Activation of ERK/MAPK signaling in osteoblasts and upregulation of Runx-2 and Osx.	Induction of osteoblast differentiation.	[50,51]
Genistein (*Genista tinctoria*)	Isoflavone	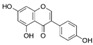	Inhibition of IL-1β-induced effects via the activation of Nrf2/HO-1 signaling in chondrocytes.	Anti-catabolic effect. Attenuated OA in pre-clinical models.	[58,59]
			Increases osteoblast differentiation via MAPK activation and inhibits osteoclast differentiation via NF-κB inhibition.	Inhibition of bone loss.	[60,61,62,63]
Icariin (*Epimedium*)	Flavonoid glycoside	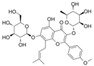	Inhibition of IL-1β/TNF-α/LPS-induced effects via the inhibition of NF-κB and the activation of Nrf2/HO-1 signaling in chondrocytes. Increases the secretion of ACAN and Col II. Decreases the expression of MMP-1, 3, 9, 13, COX-2, and iNOS.	Anti-inflammatory and anti-catabolic effects. Increased cartilage repair in pre-clinical OA models.	[64,65,66,67,68]
			Increases osteoblast differentiation via the activation of ERK, JUNK, and miR-153/Runx2 signaling. Increases the secretion of Col I APL.	Inhibition of bone loss. Improved bone remodeling in pre-clinical models.	[69,70,71,72]
Kaempferol (*Kaempferia galanga*)	3,4′,5,7-Tetrahydroxyflavone	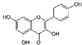	Attenuation of IL-1β-induced effects by inhibiting p38 MAPK/NF-κB pathways in chondrocytes.	Anti-inflammatory effect.	[74,75]
			Increases osteoblast differentiation via the activation of Wnt/β-catenin and mTOR signaling, increasing BMP-2, Rux-2, Osx, and Col I expression. Inhibits osteoclastogenesis by downregulating MAPK, c-Fos, and NFATc1.	Inhibition of bone loss and stimulation of bone formation.	[76,77,78,79,80,81]
Luteolin (*Salvia tomentosa*, *Artemisia asiatica*)	3,4′,5,7-Tetrahydroxyflavone	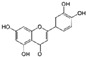	Attenuation of IL-1β-induced effects by inhibiting NF-κB pathways and the activation of Foxo3a in chondrocytes. Decreases the expression of COX-2, iNOS, MMPs, and ADAMTS-4,5. Attenuates cartilage degradation and increases Col II secretion.	Anti-inflammatory and anti-catabolic effects. Attenuation of cartilage degradation.	[83,84,85,86,87]
			Increases osteoblast differentiation via the regulation of ERK/Lrp-5/GSK-3β signaling, increasing BMP-7, Rux-2, Osx, Osc, APL, TGF-β1, and Col I expression. Inhibition of osteoclast differentiation.	Inhibition of bone loss and stimulation of bone formation.	[34,88,89,90,91,92,93,94]
Naringin (*Citrus* × *paradisi*)	Flavanone-7-O-glycoside	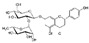	Alleviation of IL-1β/TNFα/LPS-induced effects via inhibiting MAPK p38 and NF-κB signaling and the activation of Foxo3a in chondrocytes. Decreases the expression of MMPs and ADAMTS-4,5. Attenuates cartilage degradation.	Anti-inflammatory and anti-catabolic effects. Attenuation of cartilage degradation.	[95,96,97,98]
			Increases osteoblast proliferation and differentiation. Increases the expression of Rux-2, Osx, Osc, BMP-2, OPN, and Col I expression. Inhibits osteoclast differentiation.	Inhibition of bone loss and promotes bone formation.	[99,100,101,102,103]
Puerarin (*Pueraria lobate*)	Isoflavone	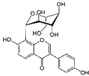	Blocks the anti-catabolic effects in chondrocytes via the action of the AMPK/PGC-1α signaling pathway. Attenuates cartilage degradation.	Anti-inflammatory and anti-catabolic effects. Attenuation of cartilage degradation.	[105,106]
			Promotes bone formation via the activation of p38 MAPK, ERK1/2-Runx2, and Wnt/β-catenin signaling and by inhibiting TRPM3/miR-204 expression. Inhibits osteoclastogenesis by downregulating CREB/PGC1β/c-Fos/NFATc1 signaling.	Inhibition of bone loss and promotes bone formation.	[107,108,109,110,111,112,113,114]
Silibinin/Silymarin (*Silybum marianum*)	Flavone	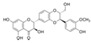	Inhibition of IL-1β-induced effects by inhibiting PI3K/Akt and NF-κB signaling. Decreases the expression of iNOS, MMPs, and ADAMTS-4,5. Diminishes the secretion of NO, PEG2, TNF-α, and IL-6. Attenuates cartilage degradation and synovitis in vivo.	Anti-inflammatory, anti-oxidant, and anti-catabolic effects. Attenuation of cartilage degradation and synovitis.	[118,119,120]
			Induces osteoblast differentiation, increasing the expression of Runx-2, BMP-2, ALP, and Col I. Inhibits osteoclastogenesis by disturbing TRAF6-c-Src signaling.	Anti-oxidant and anti-apoptotic effects in osteoblasts. Inhibition of bone loss.	[121,122,123,124,125,126]
Wogonin (*Scutellaria baicalensis*)	*O*-methylated flavone	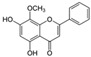	Inhibition of IL-1β-induced effects by inhibiting c-Fos/AP-1 and JAK/STAT signaling and activating ROS/ERK/Nrf2 signaling. Decreases the expression of iNOS, MMPs, and ADAMTS-4,5. Diminishes the secretion of NO, PEG2, TNF-α, and IL-6. Attenuates cartilage degradation and synovitis in vivo.	Anti-inflammatory, anti-oxidant, and anti-catabolic effects. Attenuation of cartilage degradation and synovitis.	[129,130,131,132,133,134]

**Table 3:** Natural-phenol-based pharmacology therapy for OA.

Compound	Category	Structure	Therapeutic Target	Treatment	Ref.
Curcumine (*Curcuma longa*)	Diferuloyl-methane	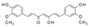	Inhibits the expression of IL-6, iNOS, COX-2, MMPs, and ADAMTS4,5 and increases the expression of SOX-9 and Col II by inhibiting 5-LOX/NF-κB signaling and activating Nrf2/ARE signaling.	Anti-inflammatory, antioxidant, anti-apoptotic, and anti-catabolic effects.	[12,135,136,137,138]
			Induces osteoblast differentiation, increasing the expression of Runx-2, Osx, Osc, and Col I by regulating Wnt signaling.	Attenuation of cartilage degradation and synovitis. Bone protection.	[139,140,141,142,143,144,145,146]
Gingerly/ginger (*Zingier officinal*)	6-Gingerol	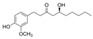	Inhibits IL-1β-induced effects via the activation of Nrf2 signaling in chondrocytes.	Anti-apoptotic, antioxidant, and anti-inflammatory effects.	[149,150]
			Induces osteoblasts differentiation and inhibits osteoclast differentiation.	Inhibition of bone loss.	[151,152]
Oleuropein (*Olea europea*)	Secoiridoid glycoside	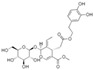	Inhibits of IL-1β-induced effects by suppressing NF-κB and MAPK signaling. Decreases the expression of COX-2, iNOS, MMP-1, MMP-13, and ADAMTS-5.	Anti-inflammatory effects. Decreases synovitis, cartilage degradation, and osteophyte formation.	[160,161]
			Increases calcium deposits and inhibits osteoclast formation and differentiation.	Bone protection.	[162,163,164,165,166]
Resveratrol (red grapes, blueberries, raspberries, mulberries)	3,5,4′-Trihydroxy-*trans*-stilbene	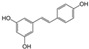	Inhibits IL-1β-induced effects by suppressing NF-κB signaling and increasing SIRT-1 expression via the AMPK/mTOR pathway. It decreases the expression of iNOS, MMP1, MMP-3, MMP-13, and ADAMTS-4,5.	Anti-inflammatory and anti-apoptotic effects. Prevents cartilage degradation and maintains the homeostasis of cartilage and bone.	[171,172,173,174,175,176,177,178,179,180]
			Induces osteoblast differentiation by modulating Sirt-1/Runx-2/Fox-1 and PI3K/AKT/mTOR signaling. Inhibits osteoclastogenesis via the activation of SIRT-1 and FoxOs.	Bone protection.	[181,182,183,184,185,186,187,188]
Salvianolic acid B (*Radix salvia miltiorrhiza*)	Polyphenol	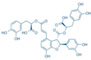	Inhibits IL-1β-induced effects by suppressing NF-κB signaling. Decreases the expression of iNOS, COX-2, MMP-13, and ADAMTS-5.	Anti-inflammatory and anti-catabolic effects. Reduces cartilage degradation.	[192,193]
			Induces osteoblast differentiation through the activation of ERK signaling, upregulating the expression of Runx-2, OPN, and Osx.	Bone protection and induces bone formation	[194,195,196,197]

**Table 4:** Natural-polysaccharide-based pharmacology therapy for OA.

Compound (Source)	Category	Therapeutic Target	Treatment	Ref.
*Achyranthes bidentata* extracts	Various polysaccharides	Induces chondrocyte proliferation, Wnt/β-catenin pathway activation, and inhibits apoptosis via MAPK/Akt signaling.	Anti-apoptotic effect and induces proliferation.	[199,200]
		Promotes bone formation and inhibits osteoclastogenesis via the inhibition of RANK.	Bone formation.	[201,202,203]

**Table 5:** Natural-terpenoid-based pharmacology therapy for OA.

Compound (Source)	Category	Structure	Therapeutic Target	Treatment	Ref.
Andrographolide (*Andrographis paniculate*)	Diterpenoid	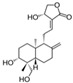	Reduces oxidative stress and inhibits MMP-13 expression. Attenuates cartilage degradation via miR-27-3p/MMP13 signaling.	Anti-oxidant effects. Reduces the degradation of cartilage.	[210,211,212,213,214,215,216,217,218,219,220,221,222]
			Promotes bone formation by inhibiting NF-κB signaling.	Bone formation	[213,214]
Astaxanthin (*Haematococcus pluvialis*)	Tetraterpenoid	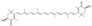	Anti-catabolic effects via the activation of Nrf2–ARE signaling. Reduces cartilage degradation via MAPK signaling inhibition.	Antioxidant and anti-inflammatory effects. Attenuates degradation of cartilage	[221,222]
Aucubin (*Aucuba japonica*)	Iridoid glycoside	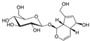	Inhibits IL-1β-induced effects. Inhibits iNOS expression and NO production	Anti-inflammatory and antioxidant effects. Prevents OA progression.	[226,227,228,229]
*Boswellia serrata*	11-Keto-β-boswellic, acetyl-11-keto-β-boswellic acid	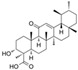	Inhibits IL-1β/oncostatin-M-induced effect, decreasing the expression of MMP-9, MMP-13, and COX-2 and reducing the production of NO and PGE2. Inhibits 5-LOX and TNF-α.	Anti-inflammatory and antioxidant effects.	[15,232,233,234]
		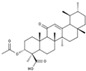	Promotes osteoblast differentiation and suppresses osteoclastogenesis by inhibiting TNF-α and NF-κB signaling.	Bone protection.	[235,236]
Celastrol (*Celastrus regelii, Tripterygium wilfordii*)	Triterpenoid	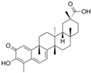	Diminishes the IL-1β-induced catabolic effect, decreasing the expression of MMP-1, MMP-3, MMP-13, iNOS, and COX-2. Reduces cartilage degradation by inhibiting NF-κB signaling and activating SDF-1/CXCR4 signaling.	Anti-inflammatory and anti-catabolic effects.	[238,239,240]
			Suppresses osteoclastogenesis by inhibiting MAPK and NF-κB signaling.	Bone protection.	[241,242]
Ginsenoside (*Panax*)	Terpenoid glycoside	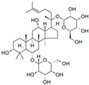	Inhibits the IL-1β-induced effect, decreasing the expression of MMP-1, MMP-13, iNOS, and COX-2; the level of PGE2; promoting the expression of ACAN and Col II.	Anti-inflammatory, anti-apoptotic, antioxidant, and anti-degradative effects.	[244,245,246,247,248,249,250,251,252,253,254]
			Promotes osteoblast differentiation and suppresses osteoclastogenesis by inhibiting MAPK and NF-κB signaling.	Bone protection	[255,256,257,258,259,260]
*Harpagophytum procumbens*	Iridoid glycosides	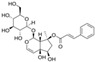	Inhibits IL-1β-induced anti-inflammatory effects, decreasing the expression of IL-6 and MMP-13 via the suppression of c-Fos/AP-1 activity.	Anti-inflammatory effect.	[263]
			Stimulates osteoblast dif-ferentiation and inhibits osteoclast differentiation.	Bone protection.	[264]
